# Diagnosis of oral potentially malignant disorders: Overview and experience in Oceania

**DOI:** 10.3389/froh.2023.1122497

**Published:** 2023-04-06

**Authors:** Alison M. Rich, Haizal M. Hussaini, Muhammad Aiman Mohd Nizar, Ratu Osea Gavidi, Elizabeth Tauati-Williams, Muhammed Yakin, Benedict Seo

**Affiliations:** ^1^ Faculty of Dentistry, University of Otago, Dunedin, New Zealand; ^2^ School of Dentistry & Oral Health, Fiji National University, Suva, Fiji; ^3^ Dental and Oral Health Services, Ministry of Health, Motootua, Samoa; ^4^ Adelaide Dental School, University of Adelaide, Adelaide, SA, Australia

**Keywords:** oral potentially malignant disorders, oral cancer, Oceania, leukoplakia, dysplasia

## Abstract

The diagnosis and management of oral potentially malignant disorders (OPMD) should be the same the world over, but there are important nuances in incidence, aetiological factors, and management opportunities that may lead to differences based on ethnogeography. In this review, we update and discuss current international trends in the classification and diagnosis of OPMD with reference to our experience in various regions in Oceania. Oceania includes the islands of Australia, Melanesia (including Papua New Guinea, Fiji, Solomon Islands, Micronesia and Polynesia (including New Zealand, Samoa, Tonga) and hence has diverse populations with very different cultures and a range from well-resourced high-population density cities to remote villages.

## Introduction

It is well accepted that advanced oral cancer is associated with worse outcomes than oral cancer diagnosed early and this is why efforts are made to detect oral cancer at an early stage, or even better, before invasion has taken place and while the lesion is still an oral potentially malignant disorder (OPMD). The prevalence of both oral cancer and OPMD varies in different geographical areas around the world and this paper will highlight regional variations relevant to Oceania. Oceania includes the islands of Australia, Melanesia (including Papua New Guinea, Fiji, Solomon Islands, Micronesia and Polynesia (including New Zealand, Samoa, Tonga). Some regions in Oceania have amongst the highest prevalence of lip and oral cavity cancer in the world (1, 2). Since oral squamous cell carcinoma (OSCC) usually progresses through a potentially malignant phase, it would be expected that the prevalence of OPMD in this region would also be high. Much less is known about the prevalence of OPMD however, as discussed below.

## Oral potentially malignant disorders

In the Chapter “Epithelial Tumours of the Oral Cavity and Mobile Tongue” the 2022 WHO Head and Neck Tumours book lists l) Papillomas 2) OPMD and Oral Epithelial Dysplasia (OED) and 3) Squamous Cell Carcinomas. The categories in OPMD and OED, on which this paper will focus, are shown in Table 1 (3).

**Table 1:** Oral potentially malignant disorders and oral epithelial dysplasia.

Oral potentially malignant disorders
Oral epithelial dysplasia
Proliferative verrucous leukoplakia
Submucous fibrosis
HPV-associated dysplasia
From Muller and Tilakaratne, 2022 (3)

The current definition of OPMD is “a heterogenous group of clinically-defined conditions associated with a variable risk of progression to oral squamous carcinoma. Most produce clinically visible lesions” (3). These clinical conditions need a biopsy to assess the degree of dysplasia, if any. When the pathologist has assessed the histology, it should be correlated with the clinical features, and an overall diagnosis can be established for the lesion at that particular point in time. The intent is that this “holistic” diagnosis provides the clinician managing the patient with the best information available to judge whether or not this lesion will be one that will eventually progress to oral squamous cell carcinoma (OSCC). It cannot be overemphasised that, while the histology is important, it has to be interpreted in light of the clinical situation, and an inadequate history and lack of clinical photographs hinder the development of a complete histological report (4). In addition, it is important to understand it is not just the site of the original lesion that needs careful review but that the entire oral mucosa may be altered and show greater susceptibility to the development of carcinoma (5).

The list of lesions currently classified as OPMDs have been published (3, 5). This paper will begin with leukoplakia- and then move on to the assessment of oral epithelial dysplasia (OED), followed by a brief discussion on oral lichen planus (OLP) and oral lichenoid lesions (OLL), the latter having been added to the list of OPMDs by the WHO Collaborating Centre for Oral Cancer in 2020. The separate categories of proliferative verrucous leukoplakia (PVL) and submucous fibrosis (SF) will also be discussed.

## Leukoplakia

Leukoplakia is “a clinical term for a white plaque of questionable risk after having excluded other known diseases or disorders that carry no increased risk for cancer” (3). It is a common condition, found to be present in about 4% of the global population (6). Overall, homogeneous leukoplakia carries only a low risk of malignant transformation but recent systematic reviews have shown a pooled transformation prevalence approaching 10% (7, 8), so full investigation and ongoing review of patients with leukoplakia are essential, if practicable. To fulfil the definition, it is important to exclude, as far as possible, a frictional cause, since frictional keratoses are essentially a hyperplastic response and as such should resolve once the initiating stimulus is withdrawn. This might involve smoothing a sharp cusp or wearing a bite splint to alter cheek or tongue chewing habits. In addition to frictional keratosis, other lesions-listed by Warnakulasuriya et al. 2020 (5) that may present as oral mucosal white patches need to be excluded before the clinical diagnosis of leukoplakia is made.

Leukoplakia is the most common OPMD; it affects more males than females and is usually seen in patients over the age of 40 years. There are geographic variations in its incidence and prevalence relating to putative aetiological factors, with a higher prevalence in South-East Asia than in Western countries (3). The prevalence of OPMDs in the Oceania region varies across the different countries. The Melanesian population, especially from Papua New Guinea and the Solomon Islands has reported a higher prevalence of white lesions (9, 10). As for the Fiji Islands, a low incidence (0.7% of the cohort) of OPMDs was found in the 35–44 year olds examined in the 2011 Fijian National Oral Health Survey (unpublished report). No OPMD were detected in a cohort of 120 Fijians of Indian descent living in Suva, Fiji (11).

In an audit of histopathology submitted to the Anatomical Pathology Laboratory, Ministry of Health in Samoa, only six OPMD specimens were received in the past 10 years and these were in patients in the age range 45–60 years (unpublished report). Data on the prevalence of OPMD in Australia and New Zealand is limited. However, a survey of nearly 6,000 oral biopsy specimens from Australian adults reported that only 1.2% of all biopsied lesions were dysplastic compared to 3.9% of biopsies from an oral pathology diagnostic service in New Zealand (12, 13).

The cause of leukoplakia appears to be linked to the same factors that cause OSCC i.e., all forms of tobacco use, betel quid and alcohol intake. Nevertheless, it is well recognised that never smokers, particularly elderly females, do develop leukoplakia, and these lesions appear to have a higher risk of malignant transformation than lesions in those with recognised risk factors (5). The prevalence of leukoplakia has repeatedly been shown to be higher in tobacco users than non-tobacco users in a range of different populations (14). The high prevalence of leukoplakia in people from Papua New Guinea and Solomon Islands is attributed to the habits of betel nut chewing, use of smokeless tobacco and tobacco smoking in these populations (9, 10). Smoking is the most common form of tobacco use in Australia and New Zealand, mainly as manufactured or roll-your-own cigarettes. In 2020–2021, one in 10 adults in Australia (10.7%) and New Zealand (10.9%) were daily tobacco smokers (15, 16). In Australia, tobacco use was higher in men (12.6%) than women (8.8%) and in people living in remote areas (19.6%). The age group with the highest tobacco use were those in their 5th and 6th decades of life. People aged 40 and older were more likely to be heavy smokers (20 or more cigarettes per day) (15, 16). A significantly higher proportion of Indigenous than non-Indigenous Australian adults were daily smokers (15). This was higher in males (45.6%) than females (41.2%). Similarly, in New Zealand, a significantly higher proportion of Māori (22.3%) and Pacific Islanders (16.4%) were daily smokers compared to the other ethnic groups in New Zealand (16). In 2011, 30.8% of 2,586 people surveyed from the Fijian population used tobacco (17) with a decline in tobacco use in both sexes and in both I-Taukei and Fijian Indians between 1980 and 2011 (18). A cross-sectional study of 120 Fijians of Indian descent living in Suva, Fiji showed that 32.5% smoked tobacco, 20% chewed betel quid or paan masala, and 14.2% chewed smokeless tobacco (11). Of 1,204 males sampled in Samoa in 2019–2020, 35.4% had smoked tobacco or used smokeless tobacco products in the preceding month, as had 12.5% of 4,139 females (19).

Less than 5% of Australian tobacco smokers used a water pipe and/or pipe tobacco, and less than 10% of current smokers reported having used e-cigarettes (15). Smokeless tobacco is relatively uncommon in Australia and New Zealand, but an increase in the number of patients presenting with lesions related to betel quid use, which frequently contains tobacco leaves, has been reported by an oral medicine specialist centre in Melbourne (20). In Australia, it is illegal to import betel quid/ areca nut, while in New Zealand, betel quid use is extremely uncommon, possibly resulting from the restrictions on its use. As we have previously reported, occasional cases of submucous fibrosis in migrants originally from the Indian subcontinent have been reported in New Zealand (21).

Most Australian adults (79%) consumed alcohol in 2019 (15). The proportion of Australians aged 14 and above who consume alcohol was 1.5 times higher in people living in remote and regional areas in 2019. Australian alcohol guidelines recommend that individuals should not consume more than 10 standard drinks a week and no more than 4 standard drinks on any given day (22). Of note, one in four Australian adults (25.8%) exceeded the Australian alcohol guidelines, particularly in the 50–59 age group (15). This trend was higher in males (33.6%) than females (18.5%) and slightly higher in Aboriginal and Torres Strait Islander people of Australia (18.7% compared with 15.2% in non-Indigenous Australians). Similarly, 20% of New Zealand adults in 2020/2021 had a hazardous drinking pattern (a pattern that “carries a high risk of future damage to physical or mental health”), with the highest rate in 18–24 year-olds (16). A significantly higher proportion of Māori (33.2%) and Pacific Islanders (26.5%) had a hazardous drinking pattern compared to other ethnic groups in New Zealand. In 2011, 41.7% of a surveyed population in Fiji indicated they had never consumed alcohol (17) whereas 15.7% said they were “current drinkers” with more males 26.1% than females 5.4%, while 32.6% of Samoan males and 4.5% of females had consumed alcohol during the previous month (19). Kava, a drink popular in Fiji and some other Pacific Islands, is made from the ground roots of the Piper plant and induces a sedative effect. It does not contain alcohol, and moderate use is not known to be associated with significant side effects (23). In a Fijian group surveyed 59% had consumed kava in the last month (78.7% of males and 38.3% of females) (17).

**Figure 1:**
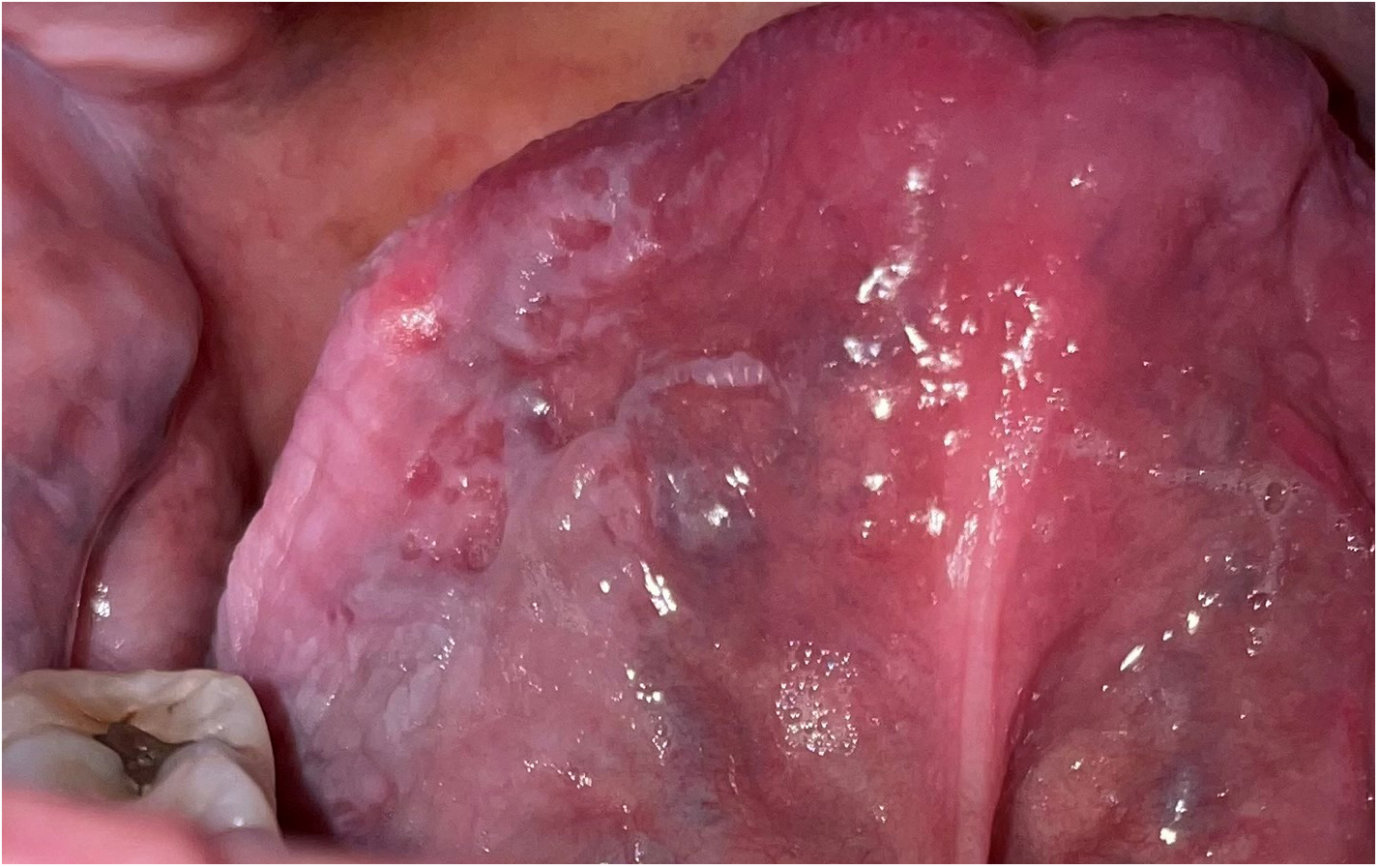
Extensive homogeneous leukoplakia involving the ventral tongue. Both its size and site are clinical indicators that this lesion is of clinical significance and has a greater potential to become malignant than a smaller leukoplakia in a different intra-oral site. It needs a base-line biopsy to enable correlation of the clinical findings with the histology findings before a treatment decision is made.

According to their clinical appearance, leukoplakias are described as homogeneous or non-homogeneous. Homogeneous leukoplakia is a predominantly white lesion which is flat with a consistent texture throughout. Most leukoplakias, (approximately 90%), are homogeneous and, by comparison with non-homogeneous leukoplakias, have a low rate of malignant transformation (24, 25). Non-homogeneous leukoplakia may be speckled white and red and may be irregularly flat, nodular, verrucous or ulcerated. Increasing erythema in an otherwise white patch is an important clinical sign, indicating the likelihood of a more serious lesion (26). Once a candidal infection has been excluded, mixed red and white lesions should be biopsied as soon as possible. Just as nonhomogeneous leukoplakia has a greater malignant transformation rate than homogeneous lesions, leukoplakia in the floor of the mouth or ventral tongue has a greater likelihood of malignant transformation than otherwise similar lesions at other intraoral sites, independent of the degree of dysplasia (27–29). However, site association with malignant transformation rates may relate to habits endemic to a particular geographic region or ethnic group:- for example, buccal mucosal OSCC is more commonly seen in those who chew betel quid (29). The size of the leukoplakia is another clinical pointer to assist with the predication of malignancy with larger lesions (>200 mm^2^) more likely to progress than smaller lesions, particularly those that spread over more than one anatomical region (30, 31) (Figure 1).

## Oral epithelial dysplasia

**Figure 2:**
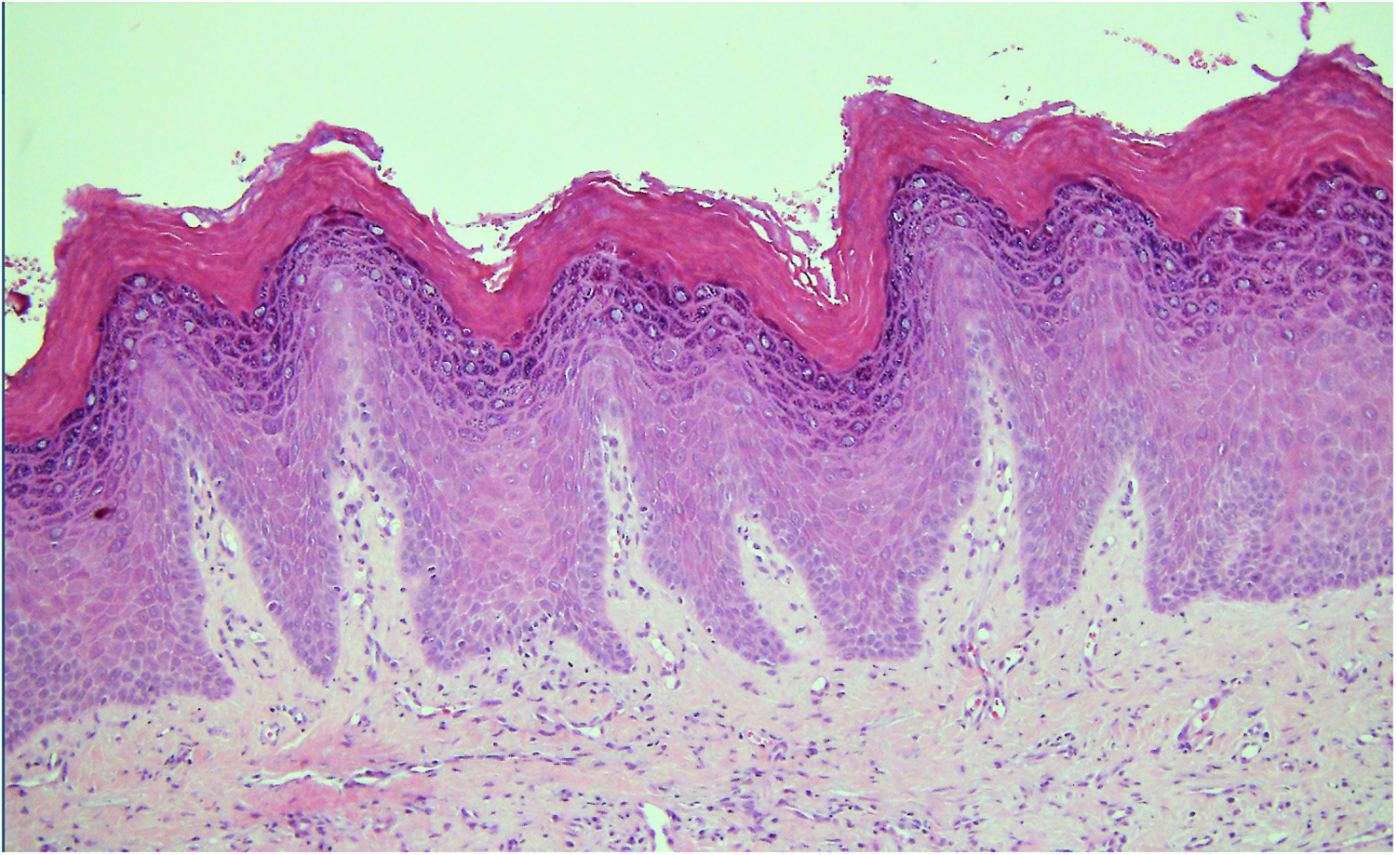
Photomicrograph from a biopsy of a white patch on the maxillary buccal gingiva in a 56 year old female. The surface epithelium is hyperplastic with a verruciform architecture. Despite the absence of cellular atypia this pattern can be associated with a higher risk of malignant transformation than a flat lesion with the same degree of cellular changes.

**Figure 3:**
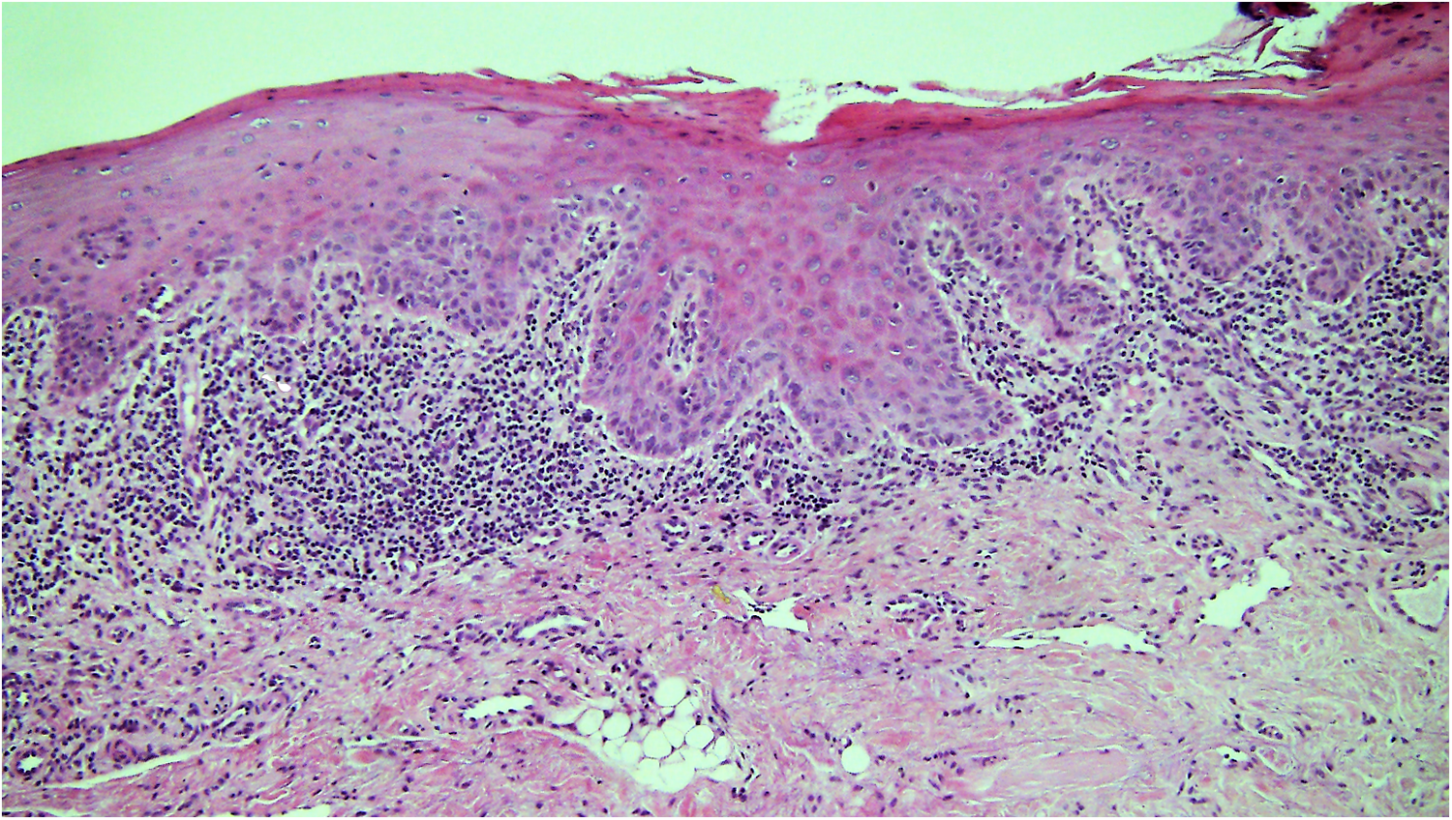
Photomicrograph from a biopsy of a white patch on the ventral tongue in a 40 year old female with a clinical provisional diagnosis of oral lichen planus. There is a band-like infiltrate of lymphocytes in the lamina propria immediately adjacent to the epithelium and some evidence of basal cell lysis, but the variability of nuclear shape and staining in the basal epithelium in addition to the rounding of the rete ridges means the epithelium is dysplastic and thus the diagnosis of lichen planus is excluded.

Histological determination of the degree of dysplasia from a biopsy of a lesion clinically diagnosed as leukoplakia is considered to be one of the most critical factors in risk assignment and determination of prognosis. Reliable assessment of OED is very important to provide the treating clinician with the information needed to make informed decisions about the management of the lesion and the whole patient. OED is “a spectrum of architectural and cytological epithelial changes resulting from accumulation of genetic alterations, usually arising in a range of OPMD and indicating a risk of malignant transformation to OSCC” (32). It results from abnormal proliferation, maturation, and differentiation of epithelial cells and it is diagnosed according to architectural and cytological disturbances (32). Architectural features include irregular epithelial stratification, drop-shaped rete ridges, reduced keratinocyte cohesion and a new addition, verrucous or papillary surface architecture. Cytological features include variations in nuclear shape, size and staining intensity and are largely unchanged. For many years, the convention to assess dysplasia has been to determine the extent of epithelial changes with changes confined to the basal third of the epithelium graded as mild dysplasia, changes extending two-thirds through graded as moderate dysplasia and full thickness as severe dysplasia. However, the reliability and reproducibility of this system have been questioned with acknowledgement of inter- and intra-observer variation (33, 34). A binary system of low-risk and high-risk lesions has been described in an attempt to improve reliability but it requires further international validation before it is fully supported. So the current recommendation is to recognise that the “thirds” system is an oversimplification but it provides a structure for the initial assessment. It should be overridden, however, in the presence of some architectural features, e.g., cytological atypia confined to the basal layer but with drop-shaped rete ridges that may be graded as severe dysplasia. Similarly, a lesion with a verrucous or papillary surface with only mild atypia may be considered to be a high-risk lesion (32) (Figure 2). Another important point highlighted in the 2022 classification is the need to recognise that a superficial lymphohistiocytic inflammatory infiltrate adjacent to the epithelium is there as an immune response to the altered basal cells, and it does not indicate oral lichen planus (OLP) or a lichenoid lesion (32, 35) (Figure 3). The presence of dysplasia in a biopsy of a lesion clinically thought to be OLP excludes the diagnosis of OLP (4, 35–37).

**Figure 4:**
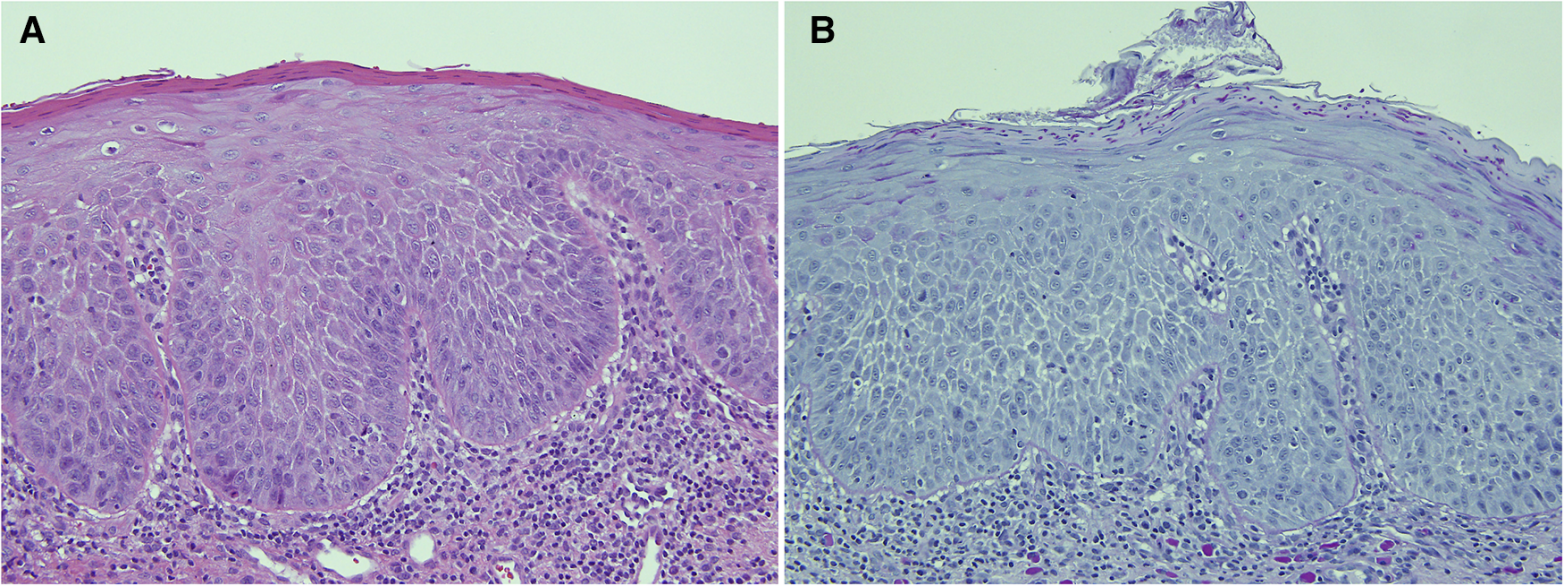
This biopsy was from a white patch on the lateral border of the tongue of a 67 year old female with a clinical provisional diagnosis of oral lichen planus. (**A**) (H&E) shows rounded rete ridges and basal cell nuclear size and shape variation with a chronic inflammatory cell infiltrate in the superficial connective tissue. The PAS-stained slide (B) shows numerous candida hyphae and yeast forms in the superficial parakeratin. The biopsy diagnosis was epithelial parakeratosis with mild dysplasia with candidal infection. The clinician was advised to re-biopsy the lesion if it persisted after a course of antifungal treatment.

Another contentious issue is the relevance of candidal infection in a biopsy of leukoplakia (Figures 4A,B). The preferred clinical term for an adherent white patch shown to be infected with *Candida* is chronic hyperplastic candidosis (CHC) rather than “candidal leukoplakia”. Chronic candidiasis has been excluded from the 2022 list of OPMD due to insufficient evidence of its malignant potential (5). It has been accepted for many years that CHC is a lesion with a risk of undergoing malignant transformation (38, 39) but the actual causative role of candidal infection in the transformation remains uncertain. We agree with the comments of Sitheeque and Samaranayake, 2003 (40), who argued that recalcitrant CHC lesions that do not resolve after antifungal therapy should be monitored closely with strong consideration given to their removal either by conventional or laser surgery.

The working assumption is that the greater the degree of dysplasia present, the greater the likelihood of malignant transformation. But most lesions with dysplasia don't progress, and the time taken for progression is variable (37). In a 10 year prospective study of 1,357 patients with biopsy-proven OPMDs in the UK, 35 (2.6%) developed OSCC (41). Patients with severe dysplasia had a greater risk of malignant transformation and earlier onset of malignancy than those with no dysplasia, independent of other variables. A similar trend was seen with lower grades of dysplasia but there was less certainty. OPMDs without evidence of dysplasia can progress to OSCC (24, 42), and the risk is a long-term one, even with low-grade dysplasia. Current treatment paradigms recommend surgical or laser excision of leukoplakia with histological evidence of high-risk dysplasia with advice regarding avoidance of known aetiological agents, particularly tobacco and alcohol, and long-term review. The management of leukoplakia with low risk or no dysplasia should also include advice about avoiding risk factors, the need for long-term review and consideration of excision of the lesion.

## Oral lichen planus and oral lichenoid lesions

Both OLP and OLL are included in the 2022 WHO classification of OPMD. There are many papers in the literature arguing whether or not OLP and OLL are potentially malignant, with the consensus that, under current criteria, OLL in particular carries a definite, but small, increased risk (3, 43).

The lack of clarity with the definitions of OLP and OLL has caused much of the controversy. It is important that the diagnosis of OLP is based on the correlation of typical clinical AND histopathological features. OLP cannot be diagnosed by histology alone and the final diagnosis should be made by the clinician, guided by the biopsy report (37, 44). The clinical and histological diagnostic criteria required for a diagnosis of OLP are described in detail by Warnakulasuriya et al. 2020 (5). The histological distinction between some cases of OLP and OED with interface mucositis can be difficult (37). Apoptotic basal keratinocytes in OLP can show various morphological changes, and the presence of intra-epithelial immune cells can make the assessment of “true” dysplasia difficult (Figure 3). For these reasons, the term lichenoid dysplasia was introduced (45), but this brought with it further complications, and the term has fallen out of favour. Currently, most pathologists agree that if a biopsy shows clear evidence of OED, OLP is excluded (35–37). OLL, defined as “oral lesions resembling lichen planus but lacking typical clinical or histopathological appearances” (3), have some features of OLP but do not comply with all the clinical or histological criteria. OLL includes atypical OLP, e.g., unilateral lesions, lesions in close proximity to a dental restoration, lichenoid drug reaction, oral lesions developing after the intake of specific substances and the oral lesions of graft vs. host disease (5, 36). Until recently, most studies assessing malignant transformation in OLP and OLL included cases with dysplasia, which is likely to have led to an over-estimate of their malignant potential (46). Still, it seems prudent to be cautious and instigate long-term review for everyone with OLP and OLL, especially those with atrophic/erosive/ulcerative forms (clinically presenting as red or ulcerated lesions) of the disease, those with lesions on the tongue and people with other risks, particularly tobacco and alcohol use (29, 47).

## Proliferative verrucous leukoplakia

The 2022 WHO classification defines proliferative verrucous leukoplakia (PVL) as a “clinico-pathological variant of oral leukoplakia that is multifocal, persistent and progressive with a high rate of recurrence, and a high risk of progression to squamous cell carcinoma” (48). It is yet another condition where a correlation between the clinical and histological features is required to make a diagnosis. It is usually a retrospective diagnosis, as the lesions evolve over time. The term PVL was introduced by Hansen et al. in 1985 (49) but- since early lesions are flat and relatively few advanced lesions are verrucous (13%–28%), some contemporary authors prefer the term proliferative leukoplakia (PL) (50, 51).

PVL is seen most often in females over the age of 60 years (51). It does not seem to be correlated with tobacco use of any form, alcohol abuse or betel quid use, and there is no apparent aetiologic association between PVL and HPV, Epstein-Barr virus or Candida albicans infection (48).

**Figure 5:**
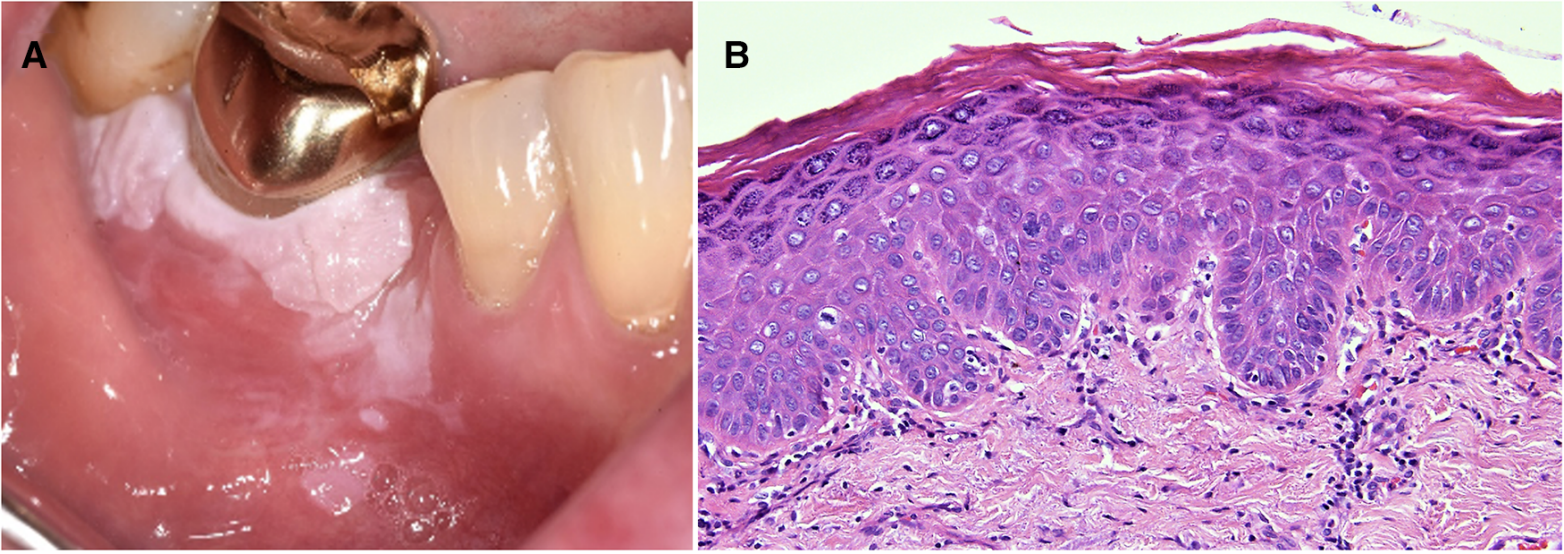
Three years prior to the development of the gingival leukoplakia pictured (**A**) this 63 year old female had an excisional biopsy of a white patch in the same region and incisional biopsies from white patches in the left and right buccal mucosa. The histology of all three lesions showed hyperkeratosis, mild dysplasia and an interface mucositis. She was under regular review with a working diagnosis of proliferative verrucous leukoplakia. The biopsy of the gingival recurrence showed epithelial hyperkeratosis with moderate dysplasia, extending to all lateral margins (**B**).

PVL evolves through a series of clinical and histological stages. Early lesions are often solitary, flat and uniformly white, sometimes with striae (Figures 5A,B). These plaques increase in size, other intra-oral sites become involved, and some become raised, non-homogeneous and verruciform. The histology reflects the clinical appearance. Early lesions will show keratosis, often without dysplasia. Lichenoid/interface mucositis features may be present in early PVL (51), and again we stress that OLP should not be diagnosed without a clear clinico-pathological correlation. The need for ongoing communication between the clinician and pathologist (and patient) when managing PVL cannot be overemphasised. Despite interventions, verrucous carcinoma and/or OSCC are the usual outcome (43, 52). Close follow-up with re-biopsy as necessary is the recommended management with laser ablation or surgical excision often associated with rapid recurrence (48).

## Submucous fibrosis

**Figure 6:**
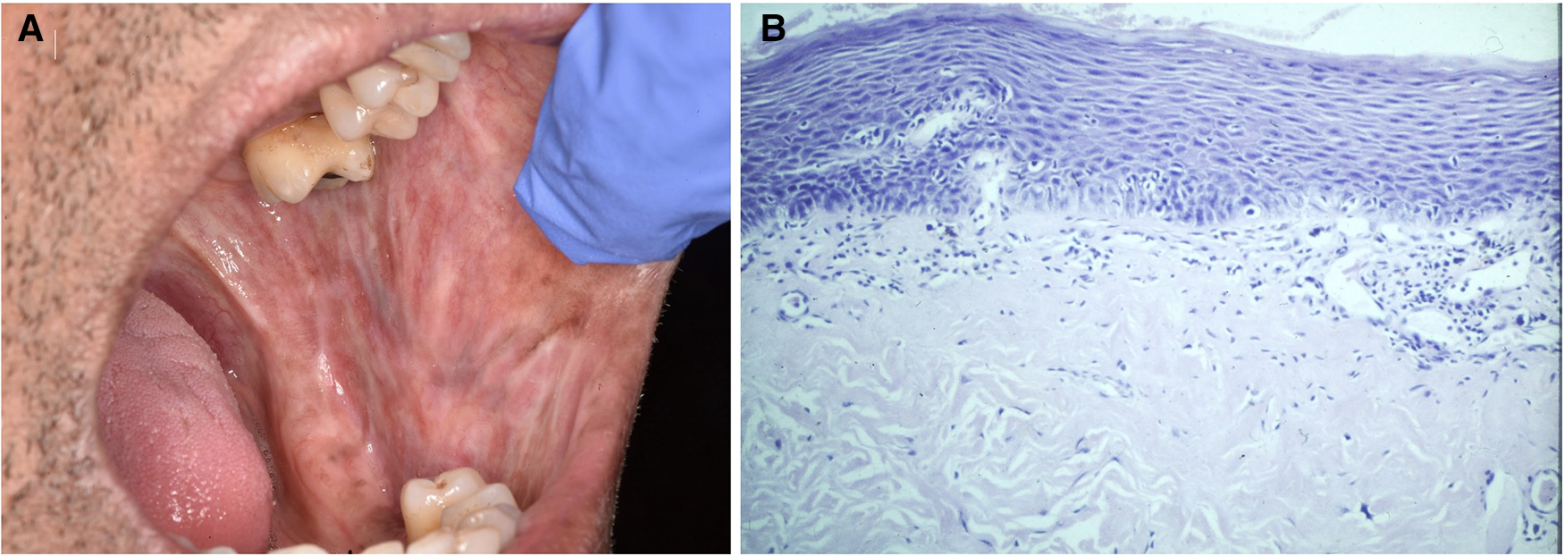
(**A**) shows oral submucous fibrosis with “marbling” of the buccal mucosa and staining of the teeth. The histology from the buccal mucosa (**B**) shows a relatively atrophic epithelium with mild dysplasia and fibrosis in the lamina propria.

“Oral submucous fibrosis (OSF) is a chronic, insidious disease characterised by progressive fibrosis of submucosal tissues of the oral cavity and the oropharynx with a risk of transformation to SCC” (53). It is common in countries where betel quid habit is prevalent i.e., South (India, Pakistan, Sri Lanka) and South-East Asia (Malaysia, Thailand, Indonesia) with the highest prevalence in Papua New Guinea, Bangladesh and India (54, 55). The clinical presentation usually begins with a stinging and burning feeling in the mouth with progression to diffuse blanching of the buccal mucosa (Figures 6A,B) and later the development of fibrous bands in the buccal mucosa and soft palate, sometimes extending into the oropharynx and upper respiratory tract. While the fibrosis produces significant signs and symptoms, there is also epithelial atrophy and a predisposition to malignant transformation in up to ∼8% of cases (56). OSF is associated with the use of betel quid, a combination of areca nut and often other ingredients enclosed in a betel leaf and placed inside the cheek, where it provides a mild euphoric effect. It is a custom deeply embedded in social and cultural practices. Both betel quid (without tobacco) and areca nut are carcinogenic (oral and oesophageal cancer), but in some cultures, tobacco is also added to the quid. The risk of oral cancer development with the use of betel quid use is synergistic with the effects of smoked tobacco and alcohol intake (54, 55).

The presence or absence of concurrent OED in SMF can be determined in a biopsy. SMF is progressive and has no effective treatment, but the cessation of betel quid use is prudent advice. Dysplastic lesions or carcinoma arising in SMF should be treated as usual.

## Conclusion

In summary, the diagnosis of OPMD relies heavily on clinical observations and routine histopathology with clinicians and pathologists using current classifications and criteria. Molecular pathology is not routinely applied to the diagnosis of this type of pathology. Most areas within Oceania have access to dental care but not necessarily to specialist oral medicine, oral pathology or oral surgery services. The number of clinicians who specialise in the management of OPMD in some countries of Oceania is shown in Table 2 (57–59). The smaller countries have a relative lack of specialised services, but even in countries like Australia, specialists are usually located in urban areas. Patients in remote areas have significant barriers to accessing care, with sometimes vast distances to travel. In any country, cost and lack of prioritisation of asymptomatic oral lesions reduce the likelihood of patients seeking ongoing care and returning for follow-up visits.

**Table 2:** Number of specialists managing OPMD in Oceania.

Country	Total population	Oral Pathologists	Oral Medicine Specialists	Oral and Maxillofacial Surgeons and Oral Surgeons
Australia	∼26 million in 2023*	20 in 2022**	44 in 2022**	292 in 2022**
New Zealand	∼5 million in 2023*	5 in 2022***	6 in 2022***	58 in 2022***
Fiji	∼901,000 in 2023*	1****	0	9
Samoa	∼201,000 in 2023	1****	0	1

* (57).

** (58).

*** (59).

**** Personal communications.

Nevertheless, enhancing awareness about the value of early detection of OPMDs to the public and clinicians remains very important. Continuing education courses suitable for all clinicians managing oral health should be available face-to-face and electronically. Access to examination and referral checklists and proformas may be helpful (60), and each centre should have clearly defined and consistent management plans. Enhancing awareness about avoidance of the preventable risk factors mentioned earlier is also crucial. A report on the evaluation of oral cancer prevention by the International Agency for Research on Cancer confirmed that cessation of tobacco smoking, alcohol consumption and the use of areca nut products had a preventive effect on the prevalence of oral cancer and also was likely to decrease OPMDs (61). Habits such as betel quid use have strong cultural significance but education programmes, such as providing Papua New Guinea's school children with information about the harmful effects of betel quid use, have been effective and are strongly encouraged (62).
